# The Use of Fragmented, Worn-Out Car Side Windows as an Aggregate for Cementitious Composites

**DOI:** 10.3390/ma12091467

**Published:** 2019-05-07

**Authors:** Maciej Szeląg, Bartosz Zegardło, Wojciech Andrzejuk

**Affiliations:** 1 Faculty of Civil Engineering and Architecture, Lublin University of Technology, 40 Nadbystrzycka Str., 20-618 Lublin, Poland; 2 Department of Quantitative Methods and Spatial Management, Siedlce University of Natural Sciences and Humanities, 2 Stanisława Konarskiego Str., 08-110 Siedlce, Poland; bart.z@wp.pl; 3 Faculty of Economics and Technical Sciences, Pope John Paul II State School of Higher Education in Biala Podlaska, 95/97Sidorska Str., 21-500 Biała Podlaska, Poland; w.andrzejuk@dydaktyka.pswbp.pl

**Keywords:** recycled glass aggregate, glass waste, recycled concrete, car side windows, cement

## Abstract

The paper describes a new model of concrete production, which contains a glass cullet. A worn-out car side window have been used for the production of recycled glass aggregate (RGA) and its properties were examined. The RGA was used in concrete as a 50% and 100% mass substitute of the traditional aggregate. Basic tests of fresh concrete mix and hardened concrete were carried out. The consistency, the air content in the concrete mix, the density of hardened concrete, water absorption, water resistance, frost resistance, and the compressive strength (after 9, 28, and 90 days) were evaluated. Composite samples were also subjected to microscopic analysis. The results showed that the RGA can be recommended as an aggregate for concretes, and the features of the RGA concrete are more favorable than those of traditional concrete. The microscopic analyses allowed us to identify the reasons for improving the properties of the RGA composites.

## 1. Introduction

Almost all branches of industry significantly interfere with the natural environment, often degrading it. In connection with this, the scientific discussions more and more often draw attention to the issue of the so-called ecological security. The priority here is proper waste management, and in particular, taking actions so that the largest possible quantities of wastes can be re-used [1,2]. This activity is not only intended to prevent the formation of unwanted waste heaps, but also aims to reduce the extraction of natural resources. The proper course of action in the field of waste management is recycling, which consists the reuse of waste raw materials in order to create new products. The best solution is the secondary use of a waste material for primary production, which means the same products. This issue, despite technical possibilities, often involves economic difficulties. If obtaining raw materials for primary production is uncomplicated and not very expensive, obtaining a waste material requires many expensive technological operations such as waste collection, selection, fragmentation, transport, etc. Entrepreneurs are reluctant to take such actions. An example of this type of industry is the glass industry, i.e., manufacturers of car windscreens and side windows. Difficulties and costs resulting from the acquisition and adaptation of recycled material discourage manufacturers from the use of worn-out products for primary production.

The solution proposed in this situation is an attempt to use wastes in other branches of industry, which would not require special technological measures to be included in a production process. An example of such industrial activity is the production of concretes. The concrete plants are located in almost all urban agglomerations, which is an asset that eliminates the costs of transporting the wastes. The introduction of glass wastes into the concrete production would, therefore, require only their crushing, which in the case of car side windows is an uncomplicated procedure. Such action would have double environmental benefits: it would reduce the amount of wastes, and at the same time it would reduce the extraction of natural aggregates.

The issue of using glass as an additive to concrete was analyzed by many researchers. Many of the results of these studies were the reason for the introduction of new technologies into industrial applications. One of them is the technology for the production and dosing of glass fibers to cementitious composites [3,4,5,6,7,8,9,10,11,12]. The research proves that due to their use, concrete is definitely more durable, plastic, and it is easier to form it into any shape. The products and architectural elements made by the GRC (Glass Reinforced Concrete) method are characterized by above-average resistance, especially to adverse external factors, including low temperatures, the sun influence, and all forms of a concrete corrosion.

Another glass additive used for the concrete production is glass powder [13,14,15,16,17,18,19,20,21,22,23,24,25]. The authors emphasize the advantages of this type of glass application for cementitious composites. Such concretes have more favorable technical parameters in the form of both a mixture and as a hardened concrete. The paper [13] presents the results of tests in which glass powder made from grinded glasses, was used as a cement substitute. Addition of the glass powder allowed for the production of concrete, which as a mixture was easily workable, and after the maturation period had a compressive strength of about 220 MPa. It allowed to state that the glass powder can react with cement components, and this additive in this form possesses pozzolanic properties.

A relatively new trend is the use of recycled glass [26,27,28,29,30,31,32,33] and ceramic wastes [34,35,36,37] in the form of a substitute for traditional aggregates. These treatments from the point of view of energy savings are the most beneficial. In the case of glass, its remelting is not required, which takes place during the production of the glass fibers. In addition, the crushing process is less energy-consuming compared to the glass powder production. The addition of glass wastes to concrete, e.g., from worn-out LCD monitors [31,32], as a substitute for sand, positively affects the properties of composites, and the concretes produced with it have better strength parameters than those produced with traditional aggregate.

The main purpose of this work was to design a new cementitious composite that would contain in its volume a glass waste obtained from worn-out side windows (vehicle origin) and to examine its basic features. The material for the tests was obtained from the car junkyard and a recycled aggregate was produced. The recycled glass aggregate (RGA) has been subjected to basic tests that are carried out for traditional aggregates. Cement composites were prepared in which the RGA was used as a 50% and 100% mass substitute of the traditional gravel aggregate (4–8 mm fraction). As a comparative composite, concrete with the same composition was prepared, but containing 100% of the traditional, i.e., sand–gravel aggregate. Basic tests of both fresh concrete mix and hardened concrete were carried out for all composites. The consistency of the fresh concrete mix, the air content in the concrete mix, the density of a hardened concrete, water absorption, water resistance, and frost resistance were tested. The compressive strength after 9, 28, and 90 days of maturation was also determined. The microscopic examination was also carried out. The novelty of the paper is the assessment of the possibility of using a waste product, such as worn-out side car windows, for the production of the recycled glass aggregate with the intention of its use in concrete.

## 2. Materials and Methods

### 2.1. Materials Used

#### 2.1.1. Binder 

The Ordinary Portland Cement CEM I 42.5N-SR3/NA (Cemex, Chełm, Poland) was used as a binder for the concretes. The cement is characterized by stable physico–chemical parameters, proper setting time, high early and final strength, low alkali content, and high resistance to aggressive chemical agents. Those are the reasons which is why it is popularly used in the production of ready-mixed concretes. The detailed values of the physico–chemical parameters of the cement are summarized in Table 1.

#### 2.1.2. Superplasticizer 

As a plasticizing admixture, the Stachement 1266 (Stachema, Świdnik, Poland) superplasticizer was used. The purpose of its use was to optimize the properties of the mixture in the aspect of the amount of water used in relation to the mix consistency. It is an admixture based on polycarboxylates and lignosulphonates with a high fluidizing effect, which is preserved for a longer period than with the usual superplasticizers. The Stachement 1266 is used for the production of ready-mixed concretes, from which it is required to maintain consistency over a long period of time. It is used for the production of high-strength concretes, monolithic concrete constructions, and industrial floors. The superplasticizer is a homogeneous liquid with a dark brown color. Its density is about 1065 kg/m^3^, pH = 4.0–6.0, dry matter content—24 ± 1.2%, chloride content—≤0.1%, and alkali content ≤0.5%.

#### 2.1.3. Traditional and Recycled Glass Aggregate 

**Figure 1:**
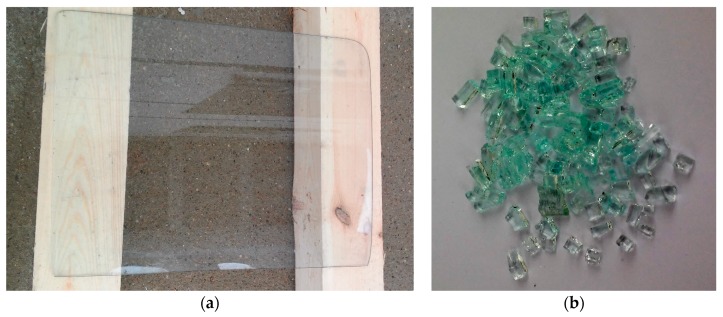
Waste glass raw material: (**a**) in the form of a solid window; (**b**) after crushing.

The raw material used to make a substitute for the mineral aggregate was car side windows coming from worn-out vehicles. The windows were taken from the scrap yard and transported to the laboratory. The material fragmentation was carried out in a jaw crusher. The windows in the first stage of crushing was broken into many small elements, which then was separated from each other. The recyclate had an irregular shape and sharp edges. Most grains had approximate dimensions of 5 × 5 × 5 mm^3^. During the crushing, a certain amount of a glass powder was also created; probably from the crushing of individual glass grains in the jaws of the crusher. Figure 1 shows a worn-out side car window and the recycled glass aggregate that has been produced.

The traditional aggregate was a sand-gravel aggregate (SGA) consisting of three fractions: 0–4 mm, 4–8 mm, and 8–16 mm. The proportions of individual fractions were determined in a computational way assuming a sand point at the level of 28%.

**Figure 2:**
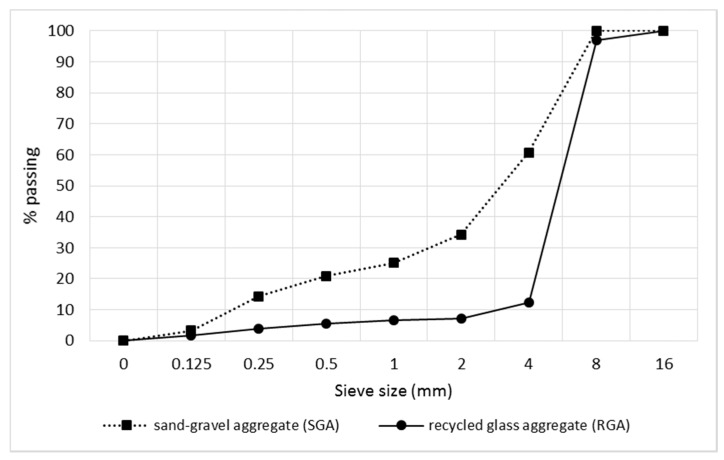
Particle size distribution curve for the recycled glass aggregate (RGA) and the traditional aggregate i.e., a sand-gravel mixture (SGA).

For the RGA and SGA, the grain size composition was assessed according to EN 933-1:2012 [38]. The particle size distribution curves for both aggregates are shown in Figure 2. The sieve analysis of the RGA showed the highest amount of the 4–8 mm fraction, which was as much as 84%. The result of this test confirmed that the particle size distribution of the RGA produced in the low-energy crushing process is not suitable, for use as a whole, as the only aggregate in the concrete composite. The grains size had too small a spread. The crumb pile would not be sealed in this case. Considering the above, it was considered the most advantageous from the point of view of the tightness of the crumb pile that the RGA should be a substitute only for the corresponding grain size of the gravel aggregate. For this reason, concrete mixes were designed, in which in the first case the RGA accounted for 50% of the substitution of the gravel aggregate of the 4–8 mm fraction, while in the second case the size of the substitute was 100%. The comparative concrete contained only the SGA.

### 2.2. Concrete Mix Design Method 

The theoretical-experimental method was used to determine the recipe of the concrete mix [39]. Designing was performed after the particle size distribution analysis of aggregates, i.e., sand of the 0–4 mm fraction, fine gravel: 4–8 mm, and coarse gravel: 8–16 mm. In order to compare the properties of concretes with RGA, a mixture was designed without the addition of the RGA, marked as the reference concrete (CONTR). When designing a concrete mix recipe, the strength class of C30/37 was established for all the concretes, which are: the reference concrete (CONTR), the concrete with 50% of the RGA (CG50), and the concrete with 100% of the RGA content in relation to the designed mass of fine gravel aggregate of the 4–8 mm fraction.

The design of the concrete mixture began with determining the qualitative features of components and determining their basic properties. On the basis of the products’ technical data sheets and own research, the bulk densities of concrete constituents, natural moisture of aggregates, and their sand points were determined. The sand point of the aggregate mixture was set at 28%. On the basis of general formulas, the proportions of aggregates were selected, followed by the amount of individual concrete components. The water/cement mass ratio (*w*/*c*) was equal to 0.39. Finally, the recipes for all concrete mixes are listed in Table 2.

### 2.3. Methodology

#### 2.3.1. Aggregates Parameters Testing 

The RGA has been tested for the basic characteristics that are being carried out for aggregates traditionally used for cementitious concrete. The SGA was also subjected to the same tests [40]. In order to determine the specific density of the aggregates, the standard method according to EN 1097-7:2011 [41] was used. The test stand has been equipped with a 50 mL pycnometer, scale, laboratory dryer, thermometer, vacuum pump, and sieve with a mesh size of 0.125 mm. 9 specific density measurements of both RGA and SGA were carried out.

The bulk density and the water absorption of the aggregate were tested using the standard method according to EN 1097-6:2011 [42]. The test stand has been equipped with a laboratory dryer, scale, water bath, laboratory sieves, drying trays, moisture absorbent cloths, stopwatch, mold with beaters, and a dryer. The values presented in the paper are the arithmetic means of six measurements for both types of the aggregates.

The strength test that was carried out for aggregates is the test of the crushing strength. The test was carried out in accordance with PN-B-06714-40:1978 [43] on an aggregate of the 4–8 mm fraction. The test stand was equipped with a container for measuring a portion of aggregate, a laboratory scale and a sieve with a mesh size of 1 mm. The strength test was performed on a hydraulic press using a special vessel with a piston for crushing the aggregate. The crushing index was determined as a percentage share of grains which after crushing passed through a sieve with a mesh size of 1 mm.

In addition, the chemical composition of RGA using the X-ray fluorescence (XRF) was examined. The test was performed on an Epsilon 3 Panalytical spectrometer (Panalytical, Almelo, The Netherlands).

#### 2.3.2. Concrete Parameteres Testing 

All concrete mixes were subjected to the consistency test according to the EN 12350-2 [44]. During all measurements, the concrete mix was laid in a cone in three layers and each time, it was compacted 25 times with a special rod. The upper surface of the element tested was carefully aligned in order to obtain the most reliable results. The measurement was made six times for each concrete mix, based on the reading of the difference in the height of the cone, and the upper surface of the concrete mix, which fell after the cone was removed.

For all concrete mixes, the measurement of air content by the Boyle–Mariotte method was also carried out [45]. The TESTING 5l porosimeter (Testing, Berlin, Germany) was used for the study. The results presented in the further part of the paper are the arithmetic mean of three measurements.

The hardened concrete has been subjected to the bulk density measurement. The test was performed on cubic samples of 100 × 100 × 100 mm^3^. Six samples of each concrete were prepared. The study was conducted in accordance with the EN 12390-7 [46].

The water absorption of concretes was also tested. The measurements were taken on identical samples as the bulk density test. Six samples of each concrete were tested. The samples were immersed in water and remained in it until their weight was constant. The water absorption was calculated in accordance with [45] as the percentage ratio of the amount of water that concrete was able to absorb to the mass of dry concrete.

The compressive strength of hardened concrete was also determined. Samples of dimensions equal to 150 × 150 × 150 mm^3^ were tested. The samples, after being molded, were subjected to the moisture care in a closed container partially filled with water. After the third day, the samples were removed from the molds, and then for the following days, they were kept in the above-described environment. The tests were carried out in accordance with the EN 12390-3 [47]. The tests involved nine samples for each concrete. The examination was carried out after 9, 27, and 90 days of maturation in optimal temperature and humidity conditions. The compressive strength tests were carried out in the laboratory on the Automax Super-Automatic EN device (Controls, Milan, Italy) from Controls.

Another test that was carried out was the assessment of the concretes’ water resistance. It was performed in accordance with the EN 12390-8:2011 [48]. This standard determines the water resistance based on the introduction of water into the sample at a pressure of 0.5 MPa and maintaining it for 72 h. During this test method, the value reflecting the water resistance is the depth of water penetration into the sample, which is measured parallel to the direction of application of the hydrostatic pressure. On the basis of the results obtained, the concrete tested can be classified only in two variants, i.e., as waterproof concrete, in the case when the water penetration depth does not exceed 50 mm, and non-waterproof concrete in the case when the water penetration depth exceeds 50 mm. The water resistance test was performed on 150 × 150 × 150 mm^3^ cubic samples. The results obtained are the arithmetic mean of six measurements.

The frost resistance of concretes was also examined. This study was carried out in accordance with [45]. It consisted of assessing the percentage decrease in the compressive strength of samples after subsequent freezing–thawing cycles. Samples were subjected to the 150 freezing–thawing cycles, which lasted 47 days. During the one cycle, the concrete was subjected to a temperature of −18 ± 2 °C for a period of 6 h, followed by thawing in water at a temperature of +18 ± 2 °C for a period of not less than 2 h. These activities were repeated until the assumed number of cycles was reached, after which the samples were subjected to a compressive strength test. The results obtained are the arithmetic mean of three samples.

The microscopic analyses were carried out using two microscopes, i.e., an optical microscope and scanning electron microscope (SEM). Samples for the SEM analysis were dried to a constant mass in a laboratory dryer. Then, they were glued to a special holder using double-sided carbon tape. In order to obtain an electro-conductive layer, the samples were subjected to the preparation consisting in the spraying of a thin layer of carbon on their surface. Observations were carried out in a high vacuum mode.

## 3. Results and Discussion

### 3.1. Evaluation of Properties of the Recycled Glass Aggregate

Table 3 summarizes the property results of the aggregates used (SGA and RGA). In addition to the parameters that were determined by the authors, the values of other features are also given based on the literature data [45].

Both SGA and RGA have very similar values, while RGA is characterized by the bulk density higher by approx. 18% compared to SGA. Thus, the structure of the traditional aggregate is more porous, which is disadvantageous, for example in the context of frost resistance or water absorption of a cementitious composite. The RGA sealed structure has a number of advantages, e.g., such an aggregate is characterized by a lower water demand, which facilitates the design of a concrete mixture, or a much higher compressive strength in relation to SGA (up to 27 times higher). In addition, very low water absorption of RGA (0.1%) will result in less water absorption of the concrete using such aggregate. High values of the RGA mechanical parameters (compressive strength and modulus of elasticity) indicate that the aggregate will be brittle at the same time. This is confirmed by the value of the crushing index, which is 4.3% higher compared to SGA.

The above-mentioned RGA features compared to SGA allow to state that the recycled glass aggregate can be successfully used for the production of concretes. In addition, glass and aggregate products obtained from it are characterized by a very high chemical resistance. Concrete made of RGA could be successfully used in an environment in which there is an increased risk of material exposure to aggressive chemical agents.

The determination of the RGA chemical composition indicated the occurrence of the following oxide equivalents: S_i_O_2_—79.6%, Al_2_O_3_—15.1%, K_2_O—3.0%, Na_2_O—1.3%, Fe_2_O_3_—0.6%, and MgO—0.4%. In the case of glass waste used no heavy metals were found.

### 3.2. Properties of Fresh Concrete Mix

The results of the consistency test are presented in Table 4. All concretes were made on the basis of the same recipe with the replacement of only one fraction of SGA with the RGA. The dosing of the amount of water and the liquefaction admixture was carried out in the same amounts and in the same way. Different results of the consistency test, with the same dosing of liquefaction components for all types of concrete proved the beneficial effect of glass cullet on this property. Glass is a material that is practically non-absorbable, therefore, it does not absorb so much water as a natural aggregate. No absorption of water through the glass cullet makes it remain between the components of the concrete, and the greater amount of available water in the cement matrix causes a greater fluidity of the mixture. It was also noticed that as the RGA content increased, the average cone precipitation also increased, i.e., the concrete mix with more glass content was more fluid. Reducing the amount of water or superplasticizer would give the possibility of obtaining the same consistency class as the concrete made entirely of traditional aggregate (CONTR). Thus, it would be possible to reduce the *w/c* ratio (which is a positive phenomenon). This translates directly to the improvement of all concrete properties, in particular, the mechanical strength.

The obtained mean results of air content in a fresh concrete mix resulted in the lack of RGA influence in this aspect. The CONTR mix has an air content of 1.80%, CG50—1.85%, and CG100—1.80%. It also proves that there are no chemical reactions between the cement matrix and the RGA grains, which would result in gas evolution.

### 3.3. Properties of Hardened Concrete

**Figure 3:**
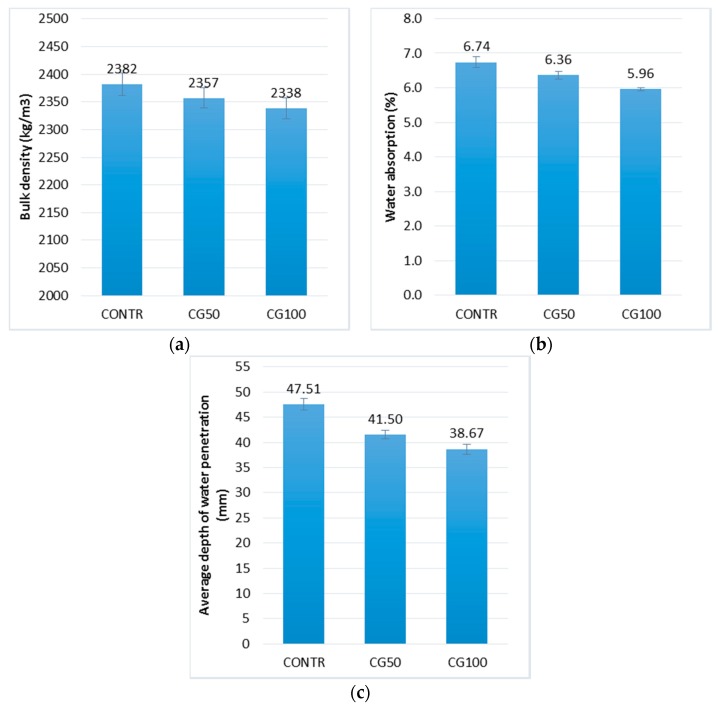
Physical properties of concrete with RGA: (**a**) bulk density; (**b**) water absorption; (**c**) water resistance (by means of average depth of water penetration); in the form of error bars, the standard deviation is shown.

Figure 3 presents the test results of physical properties of RGA concrete, i.e., bulk density, water absorption, and water resistance. The bulk density values of all concretes are very similar to each other. The difference between the concrete with the highest density (CONTR) and the concrete with the lowest density (CG100) is less than 2%. However, there is a tendency to decrease the bulk density of concrete together with the increase of the glass cullet content. The reason for this is most probably the difference in the density of the aggregates themselves and possible technological inaccuracies in the process of compacting the concrete mix.

The water absorption test confirmed the earlier assumptions regarding once less concrete absorbability with the increase of the RGA content. The reference concrete (CONTR) containing only SGA in its volume had a water absorption of 13% higher compared to the concrete with the highest content of RGA (CG100). The recycled glass aggregate is a non-absorbable and homogeneous aggregate. Gravel aggregate of the 4–8 mm fraction has an absorbability of 2.1% and it may vary depending on the content of different types of grains. The reduction in the water absorption of concrete due to the use of the RGA is another proof of the general improvement of the composite’s properties.

The water resistance test indicated that this property is more beneficial with the increase of the RGA content in the composite. The depth of water penetration for CG50 and CG100 was 14% and 22% lower, respectively, than for the CONTR. As in the case of water absorption, this is probably due to the structure of the RGA, which itself is a nonporous and nonabsorbent aggregate. This translates directly to the increase in resistance which puts the concrete composite against the hydrostatic pressure.

**Figure 4:**
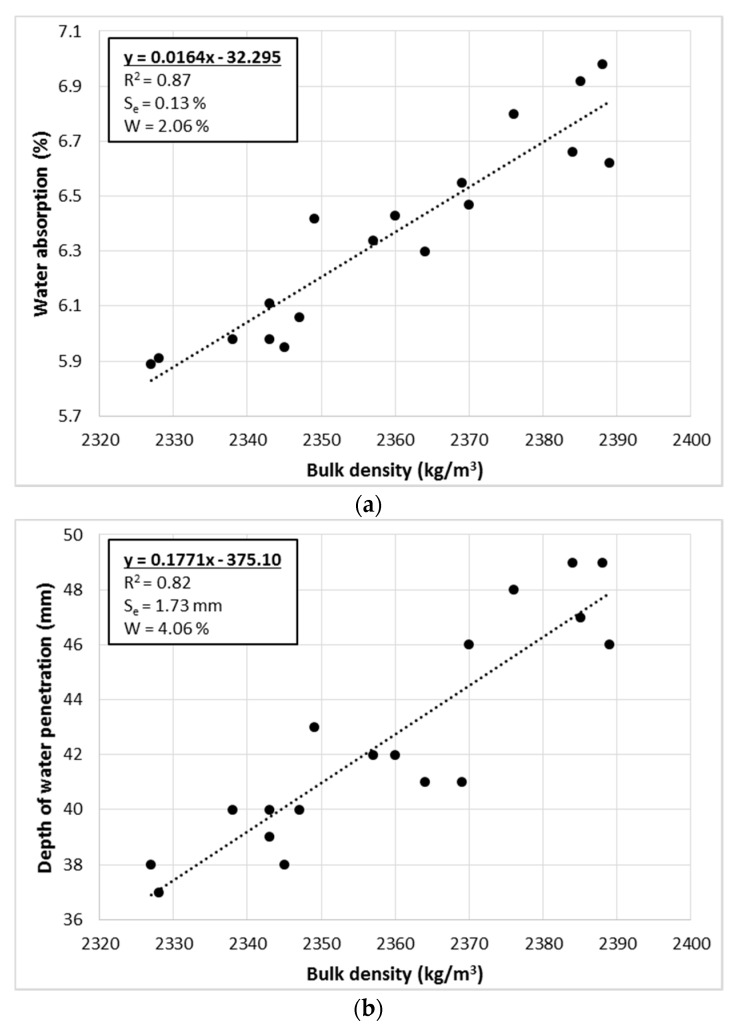

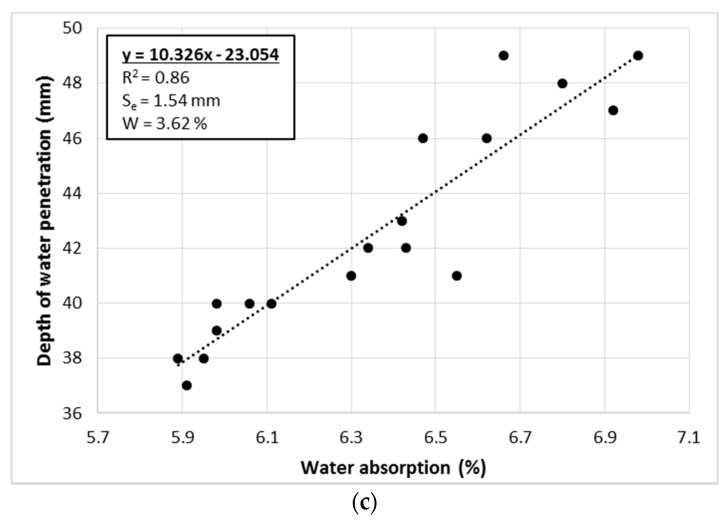
Relations between physical features of concrete with RGA: (**a**) bulk density—water absorption; (**b**) bulk density—water resistance; (**c**) water absorption—water resistance; (water resistance represented in this case by the depth of water penetration); designation of abbreviations are placed in the text.

Analyzing the entire population of results of the physical properties of concrete made with RGA, a very strong correlation was found (|*r*| > 0.9) between these parameters. The correlation coefficient between bulk density and water absorption is equal to 0.93; bulk density and water resistance (represented by depth of water penetration) −0.91; water absorption and water resistance −0.93. Figure 4 presents the relationships that occur between the physical characteristics of the cementitious composites tested for the entire population of results. The linear regression equations were calculated using the least squares method (LSM) [49,50]. Three statistical indicators were used to assess the quality of the fit of the regression lines to the empirical data: *R*^2^—the coefficient of determination, *S_e_*—the standard error of estimation, and *W*—the coefficient of random variation [51].

The linear regression equations reflect the empirical data (in each case R^2^ > 0.8). The strongest relationship is between the bulk density and water absorption, which is also confirmed by the highest value of the correlation coefficient. In addition, in this case, the risk of estimation error is very small (*W* = 2.06%). With this in mind, it is possible to calculate the water absorption of the RGA concrete based on the bulk density with very high accuracy, using the calculated linear regression equation. In the other two cases, the risk of estimation error is still low (4.06% and 3.62%), although it is almost twice as high as the relation between the bulk density and water absorption.

**Figure 5:**
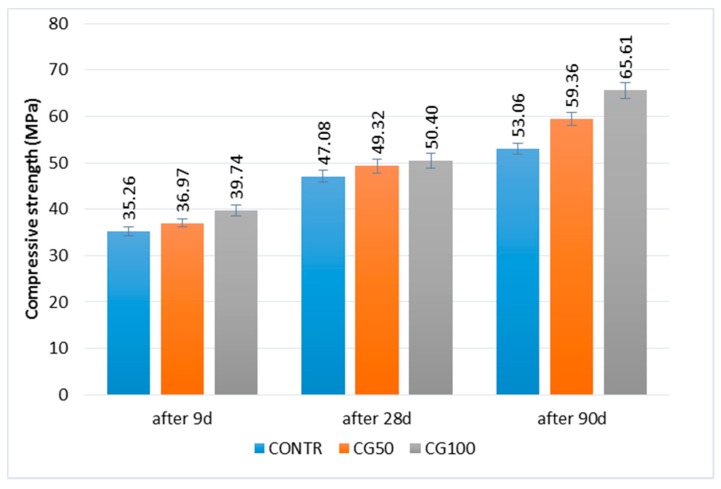
Compressive strength of concrete with RGA; in the form of error bars, the standard deviation is shown.

The compressive strength of the cementitious composites is shown in Figure 5. The analysis of the results indicated that the higher the RGA content, the higher the compressive strength is. Based on the results obtained for the reference concrete (CONTR) for the test after 9 days, it was noticed that the CG50 strength is higher by 4% and for CG100 by as much as 7%. The same comparison after 28 days of maturation indicates higher strengths also by 4% and 7%, and after 90 days by 11% and 23% for CG50 and CG100, respectively, in relation to CONTR. The increase in strength over time considered for CONTR, CG50, and CG100 was about 30% between the test after 9 and 28 days, while about 20% between the tests after 28 and 90 days. The results obtained clearly indicate the positive strength effect of the use of the RGA in concrete.

The natural aggregate has a compressive strength of approx. 30 MPa depending on the type of deposit it comes from. The glass has a compressive strength of approx. 900 MPa, thanks to which it has the ability to carry loads of much greater value. Due to the low compressive strength of the gravel aggregate, it has some limitations and, depending on the quality of the bed, it is in practice believed that it is difficult to produce cementitious composites with a strength of more than 50 MPa. The factor that can increase the compressive strength of concretes containing RGA is the grain shape (sharp, uneven edges; SGA—rounded edges and round shape). This shape unquestionably increases the adhesion of the cement matrix to the aggregate grains, whereby one of the critical spots in the concrete, i.e., the interfacial transition zone (ITZ) between the aggregate grains and the matrix, is strengthened. In the case of RGA, there is also a smaller percentage of grains in the flat and longitudinal shape, which is much more common in the case of the sand–gravel aggregate.

**Figure 6:**
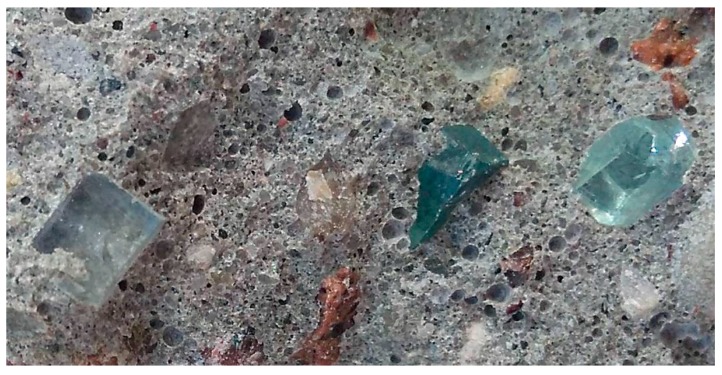
The fracture surface of the concrete sample after the compressive strength test, the undamaged RGA grains are visible.

Figure 6 presents the fracture (after compression test) of a concrete sample with RGA. Observation of the fracture proves that the destruction was caused by loss of adherence of the cement matrix to the aggregate. The RGA grains are whole, undamaged, and their fragments are projected about 3 mm beyond the face of the fracture. This confirms the high strength of the RGA itself. The test results obtained also allow to state that the contact zone of the aggregate with cement paste, despite the fact that it is usually the weakest zone in the material, in this case has a very high strength. In the upper right corner of Figure 6 there is visible a grain of gravel aggregate which was destroyed during the test (cracking through the grain). This indicates that ITZ, in the case of RGA, has a strength similar to that of the SGA grains themselves.

Table 5 presents the results of the frost resistance test of concretes with the RGA. The results indicated, as above, the positive effect of using glass cullet as an aggregate for concrete. The percentage decrease in strength after 150 freezing–thawing cycles for CONTR, CG50, and CG100 was equal to 19.6%, 8.6%, and 6.32, respectively. The decrease in strength was less and less, the more RGA was used. The glass is characterized by very high compressive strength as well as high resistance to thermal impact compared to natural aggregate. During the frost resistance test, freezing took place in an air atmosphere, while thawing took place in an aqueous environment. The water absorption of individual concretes was getting smaller as the amount of the RGA increased. Due to the lower water absorption, concretes with RGA during the thawing stage contained less water in their volume compared to CONTR. Thus, during the freezing cycle, less water has been frozen. The destructive effect of freezing water (volume increase due to the phase transition from liquid to solid) was less intensified in the case of the RGA concretes, and decreased with the increase of the RGA content. This mechanism is directly involved in the higher frost resistance of the cementitious composite with the RGA.

The material destruction due to cyclic freezing–thawing varied slightly depending on the type of aggregate used. It was noted that in many places the destruction zone ran through the coarse grains of the SGA. This is confirmed by the fact that some of the water was present in the structure of these grains during the freezing cycle. In contrast, in concretes which contained the RGA, the destruction zone ran entirely through the cement matrix. No cracking was observed in the RGA grains.

In all the tests carried out, the coefficients of variation or standard deviations were characterized by very low values. This indicates high reliability and repeatability of the results obtained, which is of great practical importance in the context of the quality of concrete with the use of the RGA.

### 3.4. Analysis of Structure and Microstructure

**Figure 7:**
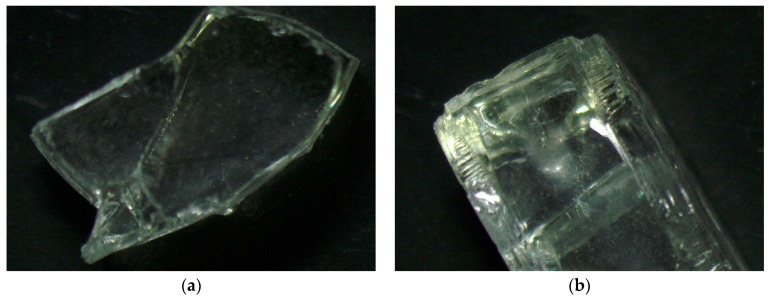

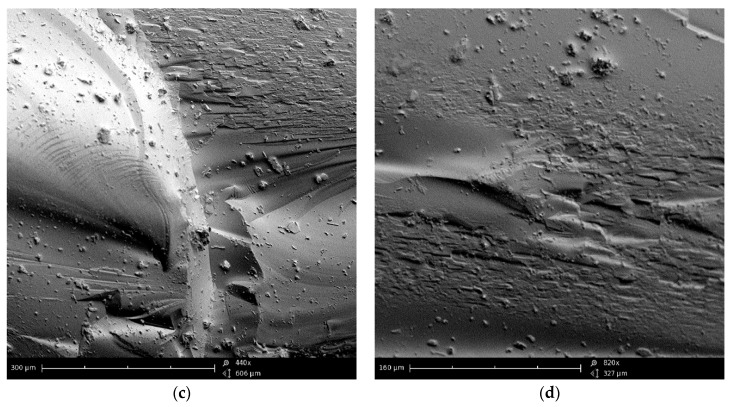
Grain of the recycled glass aggregate: (**a**,**b**) under the optical microscope; (**c**) SEM image (magnification ×440); (**d**) SEM image (magnification ×820).

Analysis of images obtained from the optical and scanning electron microscopes allowed us to assess the morphology and texture of the RGA grains as well as the concrete for which production of the RGA was used. Figure 7 shows the surface structure of the RGA grain visible to both the optical microscope and SEM. The optical microscope pictures clearly show the unevenness of the surface, which arose in the process of crushing the glass. The folds and irregularities of the aggregate increase the contact area between the cement matrix and the aggregate grains, which increases the adhesion and cohesion of the ITZ. The direct effect of this phenomenon is the increase in the mechanical strength of the RGA concrete.

Further analysis of the RGA surface morphology indicates that the individual grains consist of two types of surfaces, i.e., within one grain, a flat, smooth surface may occur on one edge, while the perpendicular surface is more uneven, sharp, and refracted. When analyzing the theoretical influence of such an aggregate grain shape on the compressive strength of the RGA concrete, it can be concluded that the strength value will depend on the direction of grain placement (orientation of individual surfaces) with respect to the direction of the compressive stresses. This is due to the fact that on the side of the smooth, flat surface of grain aggregate, the ITZ will be characterized by reduced cohesion. In addition, the force of adhesion of such a surface to the cement matrix will be reduced due to the inability of mechanical binding (formation of matrix fasteners with irregularity of the aggregate grain, which the authors have modeled, e.g., in [36]). However, the concrete mixing process results in a very random orientation of the aggregate grains with respect to the predicted compressive stresses, which finally does not affect the compressive strength achieved with the RGA concrete. This is confirmed by the very low values of the standard deviation of the results (Figure 5) that were obtained during the compressive strength test.

**Figure 8:**
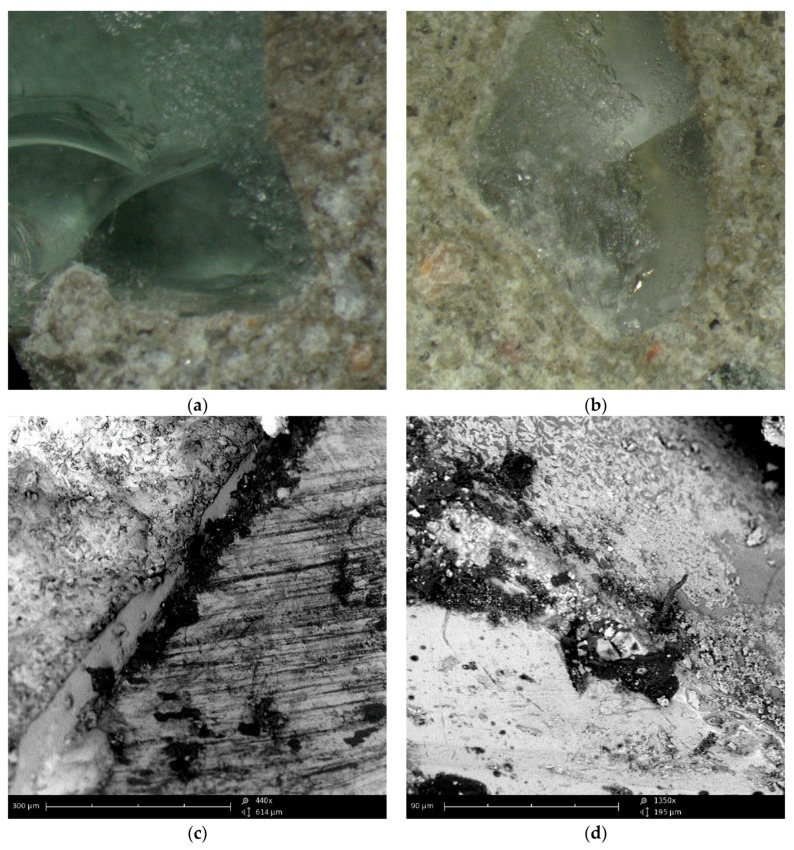
ITZ between the RGA grain and cement matrix: (**a**,**b**) under the optical microscope; (**c**) SEM image (magnification ×440); (**d**) SEM image (magnification ×1350).

**Figure 9:**
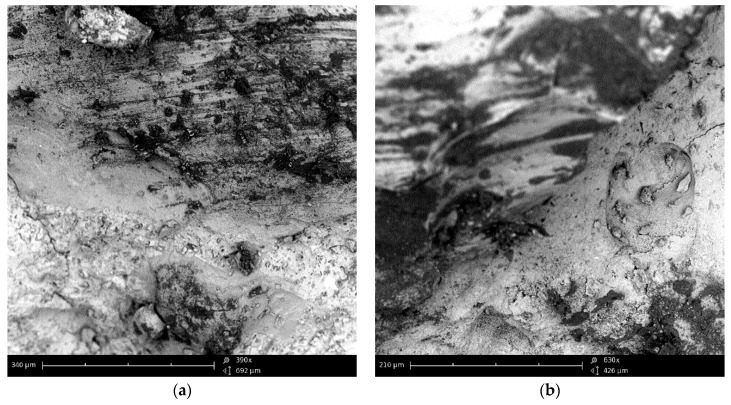
SEM image of the ITZ between the basalt aggregate grain and cement matrix: (**a**) magnification ×390; (**b**) magnification ×630.

Figure 8 shows a view of a single RGA grain that is embedded into the cement matrix after the hydration period. The contact surface of the grain with the matrix is visible, which is rough and completely filled with cement paste, which indicates a high degree of compaction. There are no cracks, delamination or cavities in it. Also, no mesopores were observed that could weaken the cohesion of the ITZ structure and, thus, degrade the properties of the final composite. Thanks to the transparency of the glass, it can be noticed that the cement paste did not penetrate inside the grain, which confirms the very low porosity of the RGA. The clearly visible boundary between the surface of the aggregate and the cement matrix also indicates the non-reactivity of the RGA grains with the components of the cement paste. The morphology of the ITZ is very similar to that observed, e.g., at the interface between cement matrix and basalt aggregate [37], which is shown in Figure 9. Due to the very high strength of RGA, the concrete damage is largely caused by the ITZ and the cement matrix itself.

Analysis of the SEM photos indicates that the basalt and RGA grains have a very similar surface structure. Both types are broken aggregates. The grain surface is sharp and ragged. Basalt grains also have a glassy surface, and cohesion between the grains and cement paste is guaranteed by adhesion forces and mechanical grip. The similarity of these features of the RGA and basalt aggregate is another proof that the glass cullet obtained from crushing car side windows is an aggregate with features more favorable than conventional mineral aggregates, i.e., sand and gravel.

**Figure 10:**
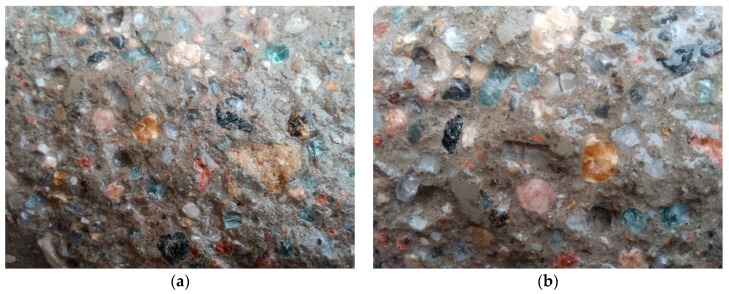
The fracture surface of the RGA concrete: (**a**,**b**) various fragments of the surface.

Figure 10 shows images of the fracture surface of the RGA concrete that have been made with a digital camera. The transparency of the aggregate grains and its high reflectivity and refraction properties increase the aesthetic value of such material compared to the conventional concrete. The use of RGA in the production of concrete facade elements and the appropriate sanding of the material surface could result in the creation of a new finishing element with improved aesthetic properties, because the light reflected from such surface causes point reflexes.

## 4. Summary and Conclusions

The paper shows the possibility of utilizing car side windows in cement composites. The solutions proposed have a beneficial effect on the technical properties of the cementitious composites. The RGA may be used in the concrete production in a local way, but not on a large scale. The aim of the production of concrete with the RGA would not be so much the production of composites with high technical values but the utilization of wastes, which are currently not effectively used.

Glass obtained from car side windows, despite the technical possibilities of its secondary use, is often deposited on landfills. This occurs mainly due to the small amount of wastes and the fact that their collection, segregation and transportation to distant places are expensive. The authors suggest to deposit car side windows at points of selective waste collection. From these sites, local concrete plants could obtain the waste and use it periodically in the case of the need to produce the cementitious composites with specific technical parameters (e.g., abrasion-resistant concrete or resistant to a chemically aggressive environment).

On the basis of the analyzed and interpreted research results, final conclusions were drawn:

- car side windows are a suitable material for the production of recycled aggregates; the particle size distribution curve of the RGA indicates that the most appropriate is its use as a 4-8 mm fraction (as a whole or partial substitute of this fraction).
- RGA has more favorable features than those of traditional mineral aggregates, i.e., sand and gravel; the aggregate is much less absorbable, has a more favorable grain shape and definitely higher strength parameters than traditional aggregates,
- cementitious concretes, in which RGA was used as a mass substitute for traditional aggregate in the amount of 50 and 100% of the 4–8 mm fraction, were characterized by lower water absorption, higher compressive strength, higher water resistance, and higher frost resistance; using the RGA, a cementitious composite was obtained with a definitely higher usable quality compared to the traditional concrete,
- microscopic analysis of RGA and concretes produced with it indicated that the structure and texture of the surface of broken glass on the edges is crystalline, uneven, and has many breaks; thus, it is similar to the surface morphology of basalt grains, which may be the reason for the greater adherence of the RGA to the cement matrix than for sand–gravel aggregates,
- the ITZ between the cement matrix and the RGA grains is cohesive, full, and has no disadvantageous features such as delamination or cavities, which increases the final cohesion and strength characteristics of the cementitious composite.

## Figures and Tables

**Table 1:** Physico–chemical parameters of the CEM I 42.5N–SR3/NA (based on the manufacturer’s data).

Feature	Unit	Average Value	Requirements
The beginning of setting time	min.	233	>60
The end of setting time	min.	291	–
Water demand	%	27.5	–
Stability of volume	mm	1.1	<10
Specific surface area	cm^2^/g	3688	–
Compressive strength: after 2 days	MPa	23.9	<10
Compressive strength: after 28 days	MPa	55.9	>42.5 <62.5
Amount of SO_3_	%	2.77	<3.0
Amount of Cl	%	0.070	<0.10
Amount of Na_2_O	%	0.53	<0.6

**Table 2:** Composition of the concrete mixes.

Component	Amount in [kg/m^3^]		
	CONTR	CG50	CG100
CEM I 42.5N-SR3/NA	337	337	337
Water	130	130	130
Sand aggregate 0–4 mm	545	545	545
Fine gravel aggregate 4–8 mm	758	379	–
Recycled glass aggregate 4–8 mm	–	379	758
Coarse gravel aggregate 8–16 mm	824	824	824
Superplasticizer: Stachement 1266	2.4	2.4	2.4

**Table 3:** Properties of recycled glass aggregate (RGA) and sand-gravel aggregate (SGA).

Feature	Unit	Sand-Gravel Aggregate (SGA)	Recycled Glass Aggregate (RGA)
Specific density	kg/dm^3^	2.65	2.62
Bulk density	kg/dm^3^	2.20	2.60
Compressive strength	MPa	33	900
Modulus of elasticity	10^2^ MPa	330	700
Water absorption	%	2.1	0.1
Crushing index	%	14.3	18.6

**Table 4:** The consistency of cementitious composite mixes.

Mix Designation	The Average Drop of the Cone [mm]	Standard Deviation [mm]	Coefficient of Variation [%]	The Consistency Class
CONTR	200	2.0	1.0	S4
CG50	220	1.8	0.8	S5
CG100	230	1.3	0.6	S5

**Table 5:** The results of frost resistance test of concrete with the RGA.

Designation of Concrete	The Average Value of the Reference Compressive Strength (MPa)	The Average Value of Compressive Strength after 150 Freezing–Thawing Cycles (MPa)	The Compressive Strength Drop after 150 Freezing–Thawing Cycles (%)
CONTR	37.27 * 0.9	31.16 2.8	19.6
CG50	48.25 1.1	44.42 3.0	8.6
CG100	60.90 2.1	57.20 2.3	6.3

* a variation coefficient value is given under the compressive strength value (%).
